# MRI-defined paraspinal muscle morphology in Japanese population: The Wakayama Spine Study

**DOI:** 10.1371/journal.pone.0187765

**Published:** 2017-11-08

**Authors:** Takahide Sasaki, Noriko Yoshimura, Hiroshi Hashizume, Hiroshi Yamada, Hiroyuki Oka, Ko Matsudaira, Hiroki Iwahashi, Kazunori Shinto, Yuyu Ishimoto, Keiji Nagata, Masatoshi Teraguchi, Ryohei Kagotani, Shigeyuki Muraki, Toru Akune, Sakae Tanaka, Hiroshi Kawaguchi, Kozo Nakamura, Akihito Minamide, Yukihiro Nakagawa, Munehito Yoshida

**Affiliations:** 1 Department of Orthopaedic Surgery, Wakayama Medical University, 811–1 Kimiidera, Wakayama City, Wakayama, Japan; 2 Department of Preventive Medicine for Locomotive Organ Disorders, 22nd Century Medical and Research Center, Faculty of Medicine, The University of Tokyo, Hongo, Bunkyo-ku, Tokyo, Japan; 3 Department of Medical Research and Management for Musculoskeletal Pain, 22nd Century Medical and Research Center, Faculty of Medicine, The University of Tokyo, Hongo, Bunkyo-ku, Tokyo, Japan; 4 Department of Orthopaedic Surgery, Faculty of Medicine, The University of Tokyo, Hongo, Bunkyo-ku, Tokyo, Japan; 5 Japan Community Health Care Organization Tokyo Shinjuku Medical Center, Shinjuku, Shinjuku-ku, Tokyo, Japan; 6 Rehabilitation Services Bureau, National Rehabilitation Center for Persons with Disabilities, 1 Namiki 4-chome, Tokorozawa City, Saitama, Japan; Leibniz-Institut fur Pflanzengenetik und Kulturpflanzenforschung Gatersleben, GERMANY

## Abstract

**Objective:**

This study aimed to establish sex- and age-dependent distributions of the cross sectional area and fatty infiltration ratio of paraspinal muscles, and to examine the correlation between paraspinal muscle degeneration and low back pain in the Japanese population.

**Methods:**

In this cross-sectional study, data from 796 participants (241 men, 555 women; mean age, 63.5 years) were analyzed. The measurement of the cross sectional area and fatty infiltration ratio of the erector spinae and multifidus from the level of T12/L1 to L4/5 and psoas major at the level of T12/L1 was performed using axial T2-weighted magnetic resonance imaging. Multivariate logistic regression analysis was used to estimate the association between fatty infiltration of the paraspinal muscles and the prevalence of low back pain.

**Results:**

The cross sectional area was larger in men than women, and tended to decrease with age, with the exception of the erector spinae at T12/L1 and L1/2 in women. The fatty infiltration ratio was lower in men than women, except for multifidus at T12/L1 in 70–79 year-olds and psoas major in those less than 50 years-old, and tended to increase with age. Logistic regression analysis adjusted for age, sex, and body mass index showed that the fatty infiltration ratio of the erector spinae at L1/2 and L2/3 was significantly associated with low back pain (L1/2 level: odds ratio, 1.05; 95% confidence interval, 1.005–1.104; L2/3 level: odds ratio, 1.05; 95% confidence interval, 1.001–1.113).

**Conclusion:**

This study measured the cross sectional area and fatty infiltration ratio of paraspinal muscles in the Japanese population using magnetic resonance imaging, and demonstrated that the fatty infiltration ratio of the erector spinae in the upper lumbar spine was significantly associated with the presence of low back pain. The measurements could be used as reference values, which are important for future comparative studies.

## Introduction

Sarcopenia, which is characterized by the loss of muscle mass and strength associated with aging, is a common problem in elderly societies [1–4]. The reduction of muscle mass and physical strength leads to disability, poor quality of life, loss of independence, and mortality [5]. The prevalence of sarcopenia in the age strata of 75–79, 80–84, and ≥85 year-olds has been reported to be 17.8%, 23.2%, and 31.8% in men and 13.8%, 22.9%, and 62.2% in women, respectively [6]. Sarcopenia is common and believed to play a major role in the pathogenesis of frailty in the aging population [1,5].

Low back pain (LBP) is also a common cause of morbidity and disability [7,8]. LBP is recognized as a multifactorial symptom. There are many causes of LBP, and the influence of paraspinal muscle degeneration on LBP has attracted interest in studies investigating the pathophysiology of LBP [9]. Muscle degeneration with aging has been characterized by muscle atrophy and fatty infiltration [10–14]. Although morphologic information on muscles can be obtained by computed tomography (CT), magnetic resonance imaging (MRI), and ultrasonic imaging techniques, MRI provides precise and reliable measurements of muscles, and can be considered the criterion standard for evaluating muscle size and structure [10–17]. Some studies reported age-related morphologic changes of the lumbar paravertebral muscles and the association between degeneration of the paraspinal muscles and LBP using MRI, but these studies included patients or volunteers, suggesting selection bias [9, 11–13]. To the best of our knowledge, no research to date has assessed age-related degeneration of paravertebral muscles in the general population.

The purpose of this study was twofold: first, to quantify age-dependent morphologic changes (muscle atrophy and fatty infiltration) of the lumbar paraspinal muscles using MRI in the Japanese population, which could be used as reference values for evaluation of the paraspinal muscles, and second, to evaluate the association between paraspinal muscle degeneration and LBP. We performed a cross-sectional, population-based study for this purpose.

## Materials and methods

This study was approved by Ethics Committee of Wakayama Medical University (No.373).

### Participants

The Wakayama Spine Study is a population-based study of degenerative spinal disease [18–22] performed in a sub-cohort of the large-scale, population-based cohort study Research on Osteoarthritis/Osteoporosis against Disability (ROAD) [23,24]. ROAD is a nationwide, prospective study of bone and joint diseases consisting of population-based cohorts established in three communities in Japan. Participants were recruited from listings of resident registrations in three communities that have different characteristics: an urban region in I town, Tokyo; a mountainous region in H town, Wakayama; and a coastal region in T town, Wakayama. Inclusion criteria, apart from residing in those communities, included the ability to walk to the survey site, to report data, and to understand and sign an informed consent form. A detailed profile of the ROAD study has already been described elsewhere [23,24]. Here, we summarize the characteristics of the present study. A third visit of the ROAD study began in 2012 and was completed in 2013. From the third visit of the ROAD study, 1575 individuals (718 individuals in the mountainous region, 857 individuals in the coastal region) were recruited to the second visit of the Wakayama Spine Study. Unfortunately, fundamental limitations allowed MRI to be conducted only in the coastal area. Thus, we evaluated data from 857 individuals in the coastal area for the present study. Among them, 42 participants with incomplete MRI records, 6 participants with unsuitable MRI for evaluating the paraspinal muscles, one participant who had previously undergone posterior lumbar fusion and 12 participants with deficits based on clinical symptoms related to LBP were excluded.

**Fig 1:**
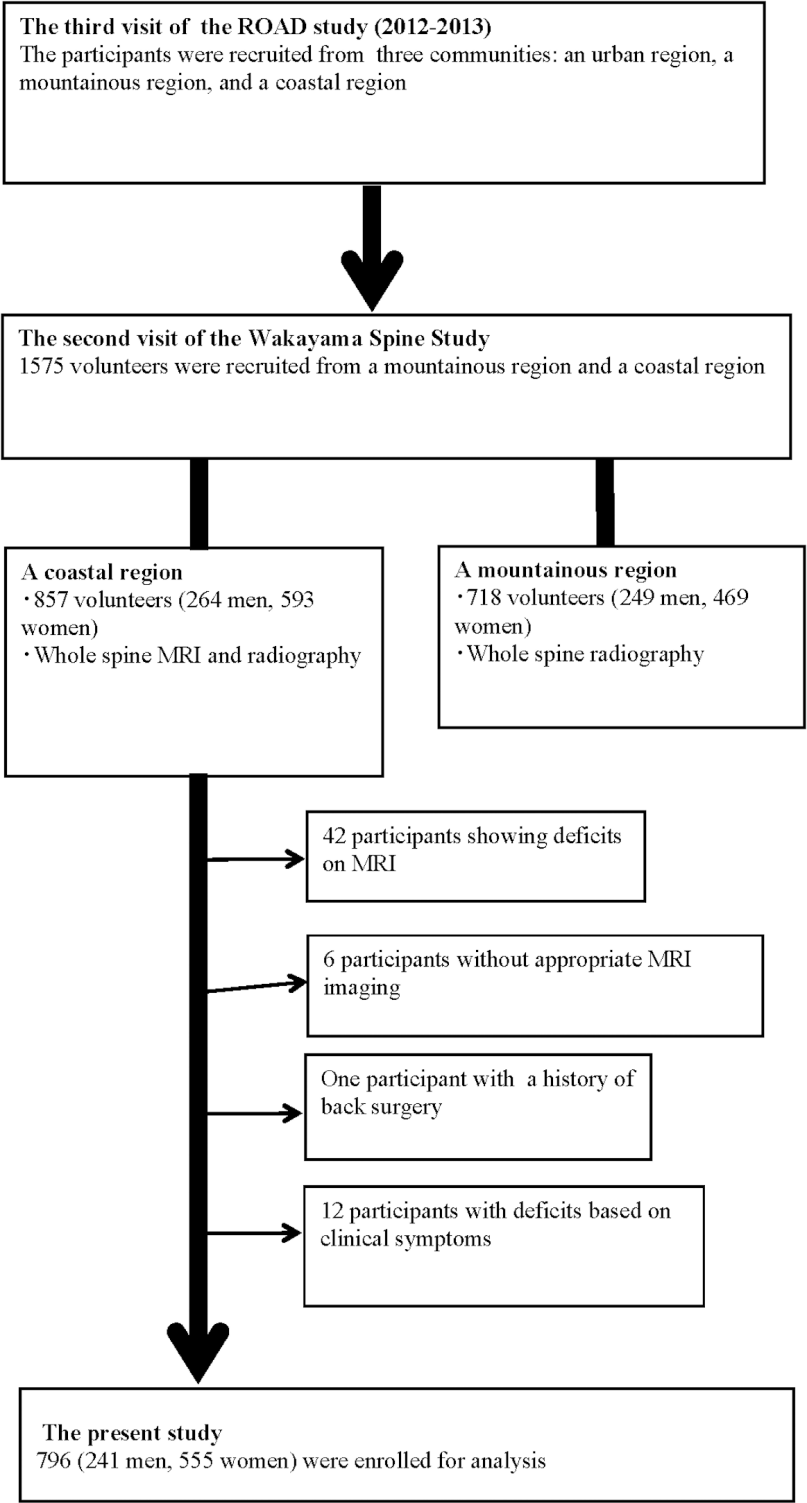
Flow diagram depicting participants recruited to the present study from the third visit of the ROAD study.

Experienced board-certified orthopedic surgeons also asked all participants the following question regarding LBP: ‘‘Have you experienced LBP on most days during the past month, in addition to now?” Those who answered ‘‘yes” were defined as having LBP based on previous studies [25–29]. We could not obtain the answer from 12 participants, therefore, these participants who lacked information regarding LBP were excluded. Thus, 796 participants (241 men and 555 women) ranging in age from 19 to 93 years-old (mean, 63.1 years-old for men and 63.7 years-old for women) were included in the analysis (Fig 1). All study participants provided informed consent, and the study design was approved by the appropriate ethics review boards.

### Magnetic resonance imaging

A mobile MRI unit (Achieva 1.5 T; Philips Medical Systems, Best, the Netherlands) was used, and whole-spine MRI was performed for all participants on the same day as the examination. The participants were supine during the MRI, and those with rounded backs used triangular pillows under their heads and knees. The imaging protocol included sagittal T2-weighted fast spin echo imaging (repetition time, 3,000 ms/echo; echo time, 120 ms; and field of view, 270 × 270 mm) and axial T2-weighted fast spin echo imaging (repetition time, 2,100 ms/echo; echo time, 100 ms; and field of view, 180 × 180 mm). Sagittal images were taken for the entire spine, but axial images were obtained for each lumbar intervertebral level (T12/L1-L5/S1) parallel to the vertebral endplates.

### Measurement of the cross sectional area and fatty infiltration ratio of paraspinal muscles

**Fig 2:**
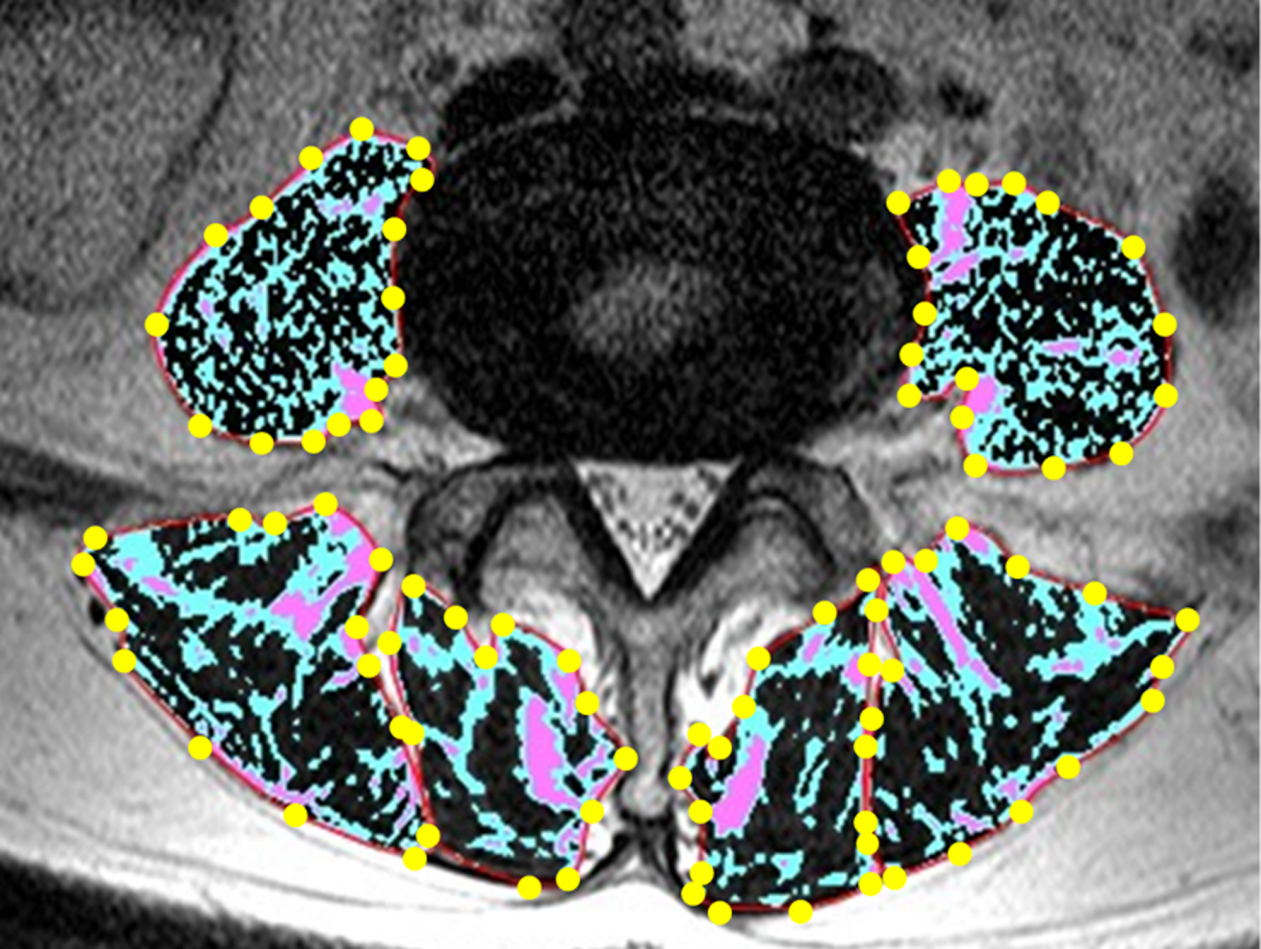
Measurement technique for CSA and FI % of paraspinal muscles. The region of black, blue, and red color represents low, medium, and high intensity areas, respectively. The high intensity area was defined as the fatty infiltration area. CSA, cross sectional area; FI %, fatty infiltration ratio.

The measurement of the cross sectional area (CSA) and fatty infiltration ratio (FI %) of paraspinal muscles (erector spinae, multifidus, and psoas major) was performed with axial T2-weighted images using a radiological workstation specially designed for such purposes. The measurement of erector spinae and multifidus was performed from the level of T12/L1 to L4/5, and that of the psoas major was performed at the level of L4/5. The CSA was measured by manually constructing polygon points around the outer margins of the individual muscles (Fig 2). The FI % was defined as the percentage of fatty infiltration area, which was obtained by dividing the fatty infiltration area by the total area. For the measurement of the fatty infiltration area, all pixels in the region of interest were sorted into three clusters based on counting pixel number and signal intensity by the k-means method [30]. That is, all pixels were distributed to low, medium, and high intensity areas. The high intensity area was defined as the fatty infiltration area (Fig 2). The CSA and FI % of paraspinal muscles were separately measured on the bilateral sides, and mean values were calculated. All measurements were taken by an orthopedic surgeon blinded to participants’ background. To evaluate inter- and intraobserver reliability for the measurement of CSA and FI %, the intraclass correlation coefficient (ICC) was calculated. To evaluate interobserver reliability, 80 randomly selected MR images were interpreted by two orthopedic surgeons (TS and HI). For evaluating intraobserver reliability, the measurements of those images were performed two times by the same observer (TS) with an interval between them greater than 1 month. All ICCs of CSA and FI % measurements were 0.99 for inter- and intraobserver reliability.

### Statistical analysis

Descriptive statistics were used to summarize demographic characteristics and the distribution of CSA and FI % of the paraspinal muscles. Wilcoxon tests were used to compare values of CSA and FI % between sexes in the corresponding group. The Jonckheere-Terpstra test was used to identify trends with regard to age for CSA and FI %. The significance of differences in demographic characteristics and FI % between groups of participants with and without LBP was tested using Wilcoxon test for continuous variables and Chi-square test for categorical data. To test the association between the presence of LBP and FI %, we used multivariable logistic-regression analysis adjusted for age, sex, and body mass index (BMI). In the regression analysis, we used the presence or absence of LBP as the objective variable, and the FI % of the erector spinae and multifidus at five disk levels (T12/L1, L1/2, L2/3, L3/4, L4/5) and psoas major at the L4/5 level, respectively, as explanatory variables, in addition to basic characteristics such as age, sex, and BMI. That is, a total of 11 models were analyzed in the multivariate logistic-regression analysis. The association between the presence of LBP and CSA was not examined because CSA was influenced by physique. All statistical analyses except the Jonckheere-Terpstra test were performed using JMP, version 12 (SAS Institute Japan, Tokyo, Japan). The Jonckheere-Terpstra test was performed using SPSS Statistics 23 (IBM Japan, Tokyo, Japan). A p-value of 0.05 was considered to indicate significant difference.

## Results

### Characteristics of participants

Table 1 shows the characteristics of the 796 participants in the present study, including age, demographic measurements, and LBP. The prevalence of LBP in men and women was 38.6% and 38.7%, respectively.

**Table 1:** Characteristics of participants.

**Men**	**Total**	**<50**	**50–59**	**60–69**	**70–79**	**≧80**
**Number of participants**	241	39	46	78	46	32
**Demographic characteristic**						
**Age (years)**	63.1±14.1	39.8±7.3	55.3±2.7	64.3±2.5	73.7±2.8	84.5±3.5
**Height (cm)**	166.7±6.8	172.4±7.0	168.4±6.4	167.3±5.1	163.5±5.6	160.8±5.5
**Weight (kg)**	66.6±10.8	72.4±8.9	67.8±11.2	68.1±10.1	64.6±10.4	57.2±7.9
**Body mass index (kg/m**^**2**^**)**	23.9±3.5	24.4±3.2	23.9±3.3	24.3±3.7	24.1±3.5	22.2±3.5
**Clinical symptoms**						
**Low back pain**	93 (38.6)	9 (23.1)	17 (37.0)	33 (42.3)	22 (47.8)	12 (37.5)
**Women**	**Total**	**<50**	**50–59**	**60–69**	**70–79**	**≧80**
**Number of participants**	555	72	123	163	145	52
**Demographic characteristic**						
**Age (years)**	63.7±12.8	41.2±6.7	54.5±2.9	64.8±2.9	74.3±2.9	83.2±2.7
**Height (cm)**	153.3±6.4	158.5±4.5	155.8±4.7	153.3±6.0	150.9±6.2	147.4±6.0
**Weight (kg)**	53.0±8.9	52.9±8.7	55.1±9.6	52.8±8.6	53.4±8.8	48.0±6.6
**Body mass index (kg/m**^**2**^**)**	22.6±3.6	21.0±3.2	22.7±4.0	22.5±3.5	23.4±3.6	22.2±3.2
**Clinical symptoms**						
**Low back pain**	215 (38.7)	24 (33.3)	49 (39.8)	60 (36.8)	55 (37.9)	27 (51.9)

Data are presented as mean ± standard deviation or as n (%).

### Distribution of CSA of paraspinal muscles

Sex- and age-dependent distributions of CSA of paraspinal muscles are shown in Table 2. According to sex, men had a significantly larger CSA in comparison to women in all muscles at all intervertebral levels and at all age strata. In terms of the effects of age on CSA, the Jonckheere-Terpstra test showed that there was a statistically significant trend towards smaller median CSAs with higher age strata in all muscles at all intervertebral levels in both genders, with the exception of the erector spinae at T12/L1 and L1/2 levels in women. The decreasing tendency of CSA of women was milder compared with that of men in all muscles at all intervertebral levels, except for multifidus at L3/4 and L4/5 levels.

**Table 2:** Distribution of CSA of paraspinal muscles (cm^2^).

**Erector spinae**	**Men**	**Total**	**<50**	**50–59**	**60–69**	**70–79**	**≧80**	**Jonckheere-Terpstra test**
	**T12/L1**	16.0±3.4*	18.5±2.7*	16.6±3.1*	16.6±2.8*	15.0±3.3*	12.5±2.9‡	p < 0.0005
	**L1/2**	18.9±3.7*	21.6±3.1*	19.3±3.3*	19.3±3.0*	17.7±3.6*	15.6±3.4*	p < 0.0005
	**L2/3**	18.4±3.6*	21.3±3.3*	18.8±3.6*	18.9±3.0*	17.2±3.0*	15.2±3.1*	p < 0.0005
	**L3/4**	16.4±3.3*	19.5±3.2*	16.7±3.2*	16.6±2.5*	14.9±2.7*	13.7±2.7*	p < 0.0005
	**L4/5**	12.8±3.0*	15.7±2.8*	13.1±3.0*	12.5±2.5*	11.4±2.3§	11.3±2.9*	p < 0.0005
	**Women**	**Total**	**<50**	**50–59**	**60–69**	**70–79**	**≧80**	**Jonckheere-Terpstra test**
	**T12/L1**	11.6±2.3	11.5±2.3	12.0±1.9	11.7±2.3	11.7±2.3	10.4±2.3	p = 0.069
	**L1/2**	13.4±2.5	13.4±2.7	13.8±2.2	13.3±2.5	13.5±2.5	12.0±2.3	p = 0.064
	**L2/3**	13.0±2.5	13.5±2.5	13.6±2.3	13.0±2.5	13.0±2.6	11.1±2.1	p < 0.0005
	**L3/4**	12.0±2.4	12.7±2.1	12.7±2.4	11.9±2.4	12.0±2.3	10.3±1.7	p < 0.0005
	**L4/5**	10.5±2.3	11.4±2.0	11.2±2.2	10.3±2.2	10.4±2.3	8.7±2.0	p < 0.0005
**Multifidus**	**Men**	**Total**	**<50**	**50–59**	**60–69**	**70–79**	**≧80**	**Jonckheere-Terpstra test**
	**T12/L1**	2.4±0.5*	2.8±0.4*	2.4±0.5*	2.4±0.5*	2.1±0.5‡	2.1±0.5‡	p < 0.0005
	**L1/2**	2.4±0.5*	2.7±0.5*	2.4±0.5*	2.4±0.5*	2.2±0.6*	2.0±0.4‡	p < 0.0005
	**L2/3**	3.3±0.9*	4.0±0.9*	3.4±0.6*	3.4±0.8*	3.1±0.8*	2.5±0.5||	p < 0.0005
	**L3/4**	5.0±1.3*	5.6±1.3*	5.3±1.0*	51±1.2*	4.6±1.5*	4.0±0.9*	p < 0.0005
	**L4/5**	7.4±1.5*	8.0±1.3*	7.8±1.4*	7.6±1.2*	7.1±1.7*	5.9±1.2†	p < 0.0005
	**Women**	**Total**	**<50**	**50–59**	**60–69**	**70–79**	**≧80**	**Jonckheere-Terpstra test**
	**T12/L1**	1.9±0.4	2.0±0.4	1.9±0.4	19±0.4	1.8±0.4	1.8±0.4	p = 0.001
	**L1/2**	1.8±0.4	2.1±0.5	1.9±0.4	1.8±0.4	1.8±0.4	1.7±0.5	p < 0.0005
	**L2/3**	2.5±0.6	2.9±0.7	2.7±0.6	2.5±0.6	2.3±0.6	2.2±0.5	p < 0.0005
	**L3/4**	3.8±0.9	4.3±0.9	4.1±0.9	3.8±0.7	3.5±0.8	3.1±0.8	p < 0.0005
	**L4/5**	5.7±1.3	6.5±1.4	6.1±1.1	5.7±1.1	5.2±1.3	4.7±1.5	p < 0.0005
**Psoas major**	**Men**	**Total**	**<50**	**50–59**	**60–69**	**70–79**	**≧80**	**Jonckheere-Terpstra test**
	**L4/5**	13.0±2.8*	15.4±2.5*	14.0±2.5*	13.1±2.4*	11.7±2.0*	9.9±1.9*	p < 0.0005
	**Women**	**Total**	**<50**	**50–59**	**60–69**	**70–79**	**≧80**	**Jonckheere-Terpstra test**
	**L4/5**	8.1±1.5	8.9±1.5	8.2±1.3	8.0±1.5	8.0±1.4	7.2±1.4	p < 0.0005

Data are presented as mean ± standard deviation. CSA, cross sectional area. The men had significantly larger CSA than the women.

* p < 0.0001

† p = 0.0004

‡ p < 0.005

§ p < 0.01

|| p < 0.05 vs. women in the corresponding group by the Wilcoxon test.

The CSAs showed a tendency to decrease with age, except for erector spinae at T12/L1 and L1/2 in women by the Jonckheere-Terpestra test.

### Distribution of FI % of paraspinal muscles

Table 3 showed sex- and age-dependent distributions of FI % of paraspinal muscles. According to sex, the women had a significantly higher FI % in comparison to the men in all muscles at all intervertebral levels at all age strata, except for multifidus at the T12/L1 level in the 70–79 years-old group and the psoas major in the less than 50 years-old group. In terms of the effects of age on FI %, the Jonckheere-Terpstra test showed that there was a statistically significant trend towards higher median FI % with higher age strata in all muscles at all intervertebral levels in both genders. The increasing tendency of the FI % of men was milder compared with that of women in all muscles at all intervertebral levels, except for the erector spinae at the L4/5 level and multifidus at the T12/L1 and L1/2 levels. According to muscle, the psoas major showed the mildest tendency towards increased FI % of all three muscles at the L4/5 level in both genders.

**Table 3:** Distribution of FI % of paraspinal muscles (%).

**Erector spinae**	**Men**	**Total**	**<50**	**50–59**	**60–69**	**70–79**	**≧80**	**Jonckheere-Terpstra test**
	**T12/L1**	6.4±2.8	4.8±2.0	5.3±1.1	6.0±1.8	8.3±4.4	8.1±2.6	p < 0.0005
	**L1/2**	6.3±2.6	5.0±2.6	5.4±1.3	5.9±1.8	8.1±3.5	7.9±2.6	p < 0.0005
	**L2/3**	6.2±2.5	4.4±1.6	5.3±1.3	6.3±2.0	7.7±3.0	7.9±2.6	p < 0.0005
	**L3/4**	7.8±2.8	5.5±2.9	6.7±1.8	7.7±2.1	9.6±2.8	10.0±2.4	p < 0.0005
	**L4/5**	10.1±3.9	6.5±1.7	8.2±2.0	10.1±3.5	12.4±3.7	14.0±3.1	p < 0.0005
	**Women**	**Total**	**<50**	**50–59**	**60–69**	**70–79**	**≧80**	**Jonckheere-Terpstra test**
	**T12/L1**	8.5±4.9*	5.5±1.2†	6.6±1.8 *	7.7±3.0*	10.9±6.4†	12.7±7.3 *	p < 0.0005
	**L1/2**	8.2±4.2*	5.5±1.3†	6.5±1.8 *	7.6±2.7*	10.2±5.1†	12.1±6.5 *	p < 0.0005
	**L2/3**	8.2±3.7*	5.5±1.3*	6.8±2.1 *	7.7±2.7*	10.2±4.0*	11.7±5.1 *	p < 0.0005
	**L3/4**	10.3±4.0*	6.7±1.5*	8.5±2.3 *	10.2±3.2*	12.4±3.8*	14.3±5.5 *	p < 0.0005
	**L4/5**	12.9±4.7*	8.3±1.7*	10.7±3.2 *	12.9±3.7*	15.5±4.5*	16.9±5.4§	p < 0.0005
**Multifidus**	**Men**	**Total**	**<50**	**50–59**	**60–69**	**70–79**	**≧80**	**Jonckheere-Terpstra test**
	**T12/L1**	11.6±4.6	7.3±1.8	9.7±3.1	11.4±3.5	15.1±5.3	15.4±3.6	p < 0.0005
	**L1/2**	9.2±4.0	5.9±2.0	7.6±2.7	8.6±2.4	12.1±4.1	13.1±4.4	p < 0.0005
	**L2/3**	7.8±3.3	5.4±1.5	6.3±1.9	7.5±2.4	10.0±4.3	10.7±3.7	p < 0.0005
	**L3/4**	7.7±3.8	5.4±2.2	6.0±2.1	7.1±2.8	9.6±4.8	11.8±3.9	p < 0.0005
	**L4/5**	8.7±4.4	5.8±1.6	7.1±2.2	7.9±3.2	11.2±4.6	13.4±6.1	p < 0.0005
	**Women**	**Total**	**<50**	**50–59**	**60–69**	**70–79**	**≧80**	**Jonckheere-Terpstra test**
	**T12/L1**	14.2±4.9*	9.8±3.0*	12.4±3.4*	14.1±3.8*	16.3±4.8¶	18.6±6.5||	p < 0.0005
	**L1/2**	11.8±5.0*	7.6±2.2*	9.7±3.2*	11.5±3.7*	14.0±5.3||	17.3±5.8†	p < 0.0005
	**L2/3**	10.6±5.3*	6.6±2.1‡	8.4±3.1*	10.0±3.6*	13.4±5.7*	15.9±7.8 *	p < 0.0005
	**L3/4**	11.4±5.6*	6.5±1.9†	9.1±3.3*	11.0±3.8*	13.9±5.4*	17.5±8.6 *	p < 0.0005
	**L4/5**	13.7±6.2*	7.9±2.2*	11.3±3.9*	13.3±4.7*	16.4±6.2*	20.9±8.5 *	p < 0.0005
**Psoas major**	**Men**	**Total**	**<50**	**50–59**	**60–69**	**70–79**	**≧80**	**Jonckheere-Terpstra test**
	**L4/5**	8.7±3.0	8.3±4.2	8.8±3.9	8.4±2.4	8.9±2.1	9.8±2.0	p < 0.0005
	**Women**	**Total**	**<50**	**50–59**	**60–69**	**70–79**	**≧80**	**Jonckheere-Terpstra test**
	**L4/5**	9.9±2.7*	8.0±1.6**	9.3±2.4||	10.2±2.6*	10.7±2.9†	10.7±2.3||	p < 0.0005

Data are presented as mean ± standard deviation. FI %, fatty infiltration ratio. Women had a significantly higher FI % than men except for multifidus at the T12/L1 level in 70–79 year-olds and the psoas major in less than 50 year-olds.

* p < 0.0001

† p < 0.0005

‡ p < 0.001

§ p < 0.01

|| p < 0.05

¶ p = 0.12

** p = 0.21 vs. men in the corresponding group by the Wilcoxon test.

FIs had a tendency to increase with age according to the Jonckheere-Terpestra test.

### Association of FI % of paraspinal muscles with LBP

The differences in demographic characteristics and FI % between participants with and without LBP are shown in Table 4. Concerning demographic characteristics, age was higher in the group with LBP than in the group without LBP. For the FI % of paraspinal muscles, the FI % of the erector spinae at T12/L1, L1/2, L2/3, and L3/4 levels and multifidus at T12/L1, L1/2, L3/4, and L4/5 levels were higher in the group with LBP (p < 0.05). The FI% of psoas major was not different between the LBP+ and LBP- groups.

**Table 4:** Differences in demographic characteristics and FI % between groups of participants with and without LBP.

	LBP +	LBP -	p-value
**No. of participants**	308	488	
**Demographic characteristic**			
**Gender female/male**	215/93	340/148	0.968
**Age (years)**	64.9±12.5	62.6±13.5	0.014
**Height (cm)**	157.5±9.3	157.3±8.8	0.857
**Weight (kg)**	57.7±10.9	56.8±11.6	0.238
**Body mass index (kg/m**^**2**^**)**	23.2±3.6	22.8±3.7	0.132
**FI % of erector spinae (%)**			
**T12/L1**	8.3±5.4	7.5±3.7	0.01
**L1/2**	8.2±4.7	7.3±3.2	0.002
**L2/3**	8.1±4.1	7.3±3.0	0.003
**L3/4**	10.0±4.2	9.4±3.6	0.038
**L4/5**	12.5±4.9	11.9±4.4	0.06
**FI % of multifidus (%)**			
**T12/L1**	13.9±5.0	13.2±4.7	0.045
**L1/2**	11.6±5.1	10.8±4.6	0.019
**L2/3**	10.3±5.3	9.6±4.7	0.06
**L3/4**	10.8±5.5	10.0±5.3	0.043
**L4/5**	12.9±6.8	11.9±5.8	0.02
**FI % of psoas major (%)**			
**L4/5**	8.4±2.5	8.5±2.6	0.568

Data are presented as mean ± standard deviation or as n. LBP, low back pain; FI %, fatty infiltration ratio

On multivariable logistic regression analysis adjusted for sex, age, and BMI, a significant association between FI % of the erector spinae at the L1/2 and L2/3 levels and LBP was identified, as shown in Table 5 (L1/2 level: odds ratio, 1.05; 95% confidence interval, 1.005–1.104; L2/3 level: odds ratio, 1.05; 95% confidence interval, 1.001–1.113).

**Table 5:** Association between FI % of paraspinal muscles and LBP on multivariable logistic regression analysis.

Explanatory variable	OR	95% CI	p-value
**FI % of erector spinae (%)**			
**T12/L1**	1.03	0.996–1.076	0.079
**L1/2**	1.05	1.005–1.104	0.03
**L2/3**	1.05	1.001–1.113	0.045
**L3/4**	1.02	0.972–1.071	0.413
**L4/5**	1.01	0.973–1.055	0.53
**FI % of multifidus (%)**			
**T12/L1**	1.02	0.981–1.054	0.366
**L1/2**	1.02	0.984–1.06	0.268
**L2/3**	1.01	0.977–1.05	0.488
**L3/4**	1.01	0.979–1.048	0.453
**L4/5**	1.02	0.991–1.052	0.173
**FI % of psoas (%)**			
**L4/5**	1	0.951–1.058	0.895

Data were obtained via multivariable logistic regression analysis, after adjustment for age, sex, and body mass index. FI %, fatty infiltration ratio; LBP, low back pain; OR, odds ratio; CI, confidence interval

## Discussion

We quantified CSA and FI % of the paraspinal muscles (erector spinae, multifidus, and psoas major) using MRI in 796 men and women aged 19–93 years-old, and examined the association between the presence of LBP and FI %. To the best of our knowledge, this is the first large-scale, population-based study to examine the sex- and age-dependent distributions of CSA and FI % in paraspinal muscles (erector spinae, multifidus, and psoas major). These changes may reflect the normal aging process, which is important as a reference for future comparative studies.

Little is known about normative degeneration of the lumbar paraspinal muscles, and comparisons with previous papers are limited. Only a few studies have examined age-related distributions in lumbar paraspinal muscle size, and these studies have reported inconsistent findings [11–13]. Takayama et al. investigated CSA of the paravertebral muscle using axial T2-weighted MRI in 160 patients aged 10 to 88 years-old (10 male and 10 female participants in each decade) with lumbar lordosis of more than 20°. CSA of the paravertebral muscle was defined by manually tracing the fascial boundary of the multifidus and erector spinae; they reported that CSA of the paravertebral muscle tended to decrease with age [11]. In agreement with this study, our investigation showed a tendency toward decreased CSA of the paraspinal muscles (erector spinae, multifidus, and psoas major) with age. On the other hand, Crawford et al. examined the volume of the erector spinae and multifidus by 2-point Dixon 3T MRI in 80 healthy volunteers aged 20 to 62 years-old (10 male and 10 female participants in each decade, one individual per sex older than 60 years of age), and they reported that muscle volume was age-independent [12]. The discrepancy among studies may be due to methodologic differences in the measurement techniques (CSA versus volume), defined paravertebral regions of interests, and study samples.

In terms of muscle composition, a few imaging studies have analyzed fatty infiltration in the paraspinal muscles; they reported the presence of age-dependent progressive fatty infiltration [11,12,14]. Lee et al. investigated fatty infiltration of the paraspinal muscles (erector spinae, multifidus, and psoas major) using CT in 650 patients without lumbar spinal symptoms who underwent CT of the abdomen and pelvis, and reported that there was a tendency toward progressive increase in fatty infiltration of the paraspinal muscles with age [14]. Our study showed an increasing tendency of FI % of the paraspinal muscles with age; this finding agrees with the above-referenced reports.

We found an age-related decrease in CSA and increase in FI % of the paraspinal muscles in the Japanese population, suggesting progressive muscle atrophy and worsening of muscle quality as a part of the normative aging process. There are some plausible explanations behind the occurrence of muscle degeneration with aging. Immobility [31], reduced nutrition [31], denervation [32], inflammation [31,33], reduced levels of and responsiveness to growth hormone, androgens, and insulin-like growth factor I [31,34–37], increased apoptosis [31,38], impaired autophagy [31,39], and mitochondrial dysfunction [31,40] have been reported as the mechanisms of muscle degeneration. There are multiple reasons for muscle degeneration, but the pathophysiologic mechanism is poorly understood. Further investigations are warranted to clarify the mechanism for muscle degeneration with aging.

We showed that men had a larger CSA than women and women had a higher FI % than men in the paraspinal muscles. These findings agree with previous studies [11,12]. In terms of differences between sexes, it is interesting that CSA of women showed a more mild decrease with age than that of men, and FI % of men showed a more mild decrease with age compared to that of women. This finding may indicate that the degeneration of the paraspinal muscles with aging tends to occur quantitatively in men and qualitatively in women.

In the present study, different patterns of age-dependent degeneration were identified in the three muscles examined (erector spinae, multifidus, and psoas major). The psoas major showed the least fatty infiltration. This finding is similar to that of a previous study. Lee et al. reported that fatty infiltration in the psoas major was minimal, unlike the erector spinae and multifidus [14]. It could be hypothesized that the psoas major is likely unaffected by age-dependent degeneration.

Several studies reported the association between the size and fat content of the paraspinal muscles and LBP [9,41,42]. Fortin et al. reviewed studies evaluating paraspinal muscle morphology in patients with LBP and control patients, and reported that paraspinal muscles were significantly smaller in patients with chronic LBP than in control patients [9]. Teichtahl et al. investigated LBP, CSA, and fatty infiltration of the erector spinae and multifidus using MRI in 72 community-based individuals; they reported that fatty infiltration of the paraspinal muscles was associated with LBP while the CSA was not [41]. In agreement with these studies, our investigation showed an association between FI % of the erector spinae and LBP. However, only the only erector spinae was associated with LBP, and the intervertebral levels showing a correlation between FI % and LBP were only L1/2 and L2/3 on multiple logistic regression analysis, despite the presence of fatty degeneration of all three muscles at all intervertebral levels from T12/L1 to L4/5. In addition, the odds ratio was 1.05, which is not high. These findings indicate that degeneration of the paraspinal muscles does not directly cause LBP. Although the age-related degeneration of paraspinal muscles might not strongly correlate with LBP, these findings contribute to our understanding of LBP.

The present study has several limitations. First, the CSA and FI% of paraspinal muscles was measured using in-house developed software, which is not validated externally. However, the image analysis method of our software is consistent with that of previous studies. In other words, visible storage of lipids in adipocytes underneath the deep fascia of muscle, which includes the visible storage of lipids in adipocytes located between the muscle fibers and also between muscle groups, was detected as fatty infiltration of muscles [43]. Moreover, the intra-class correlation coefficient of our method was substantially high for inter- and intra-observer reliability. Thus, we believe that our image analysis method is reliable to detect the fatty infiltration of muscles. However, the smaller group of lipids stored within the muscle cells, which are known as intramyocellular lipids, could not be detected in the present study [43]. This is a major limitation of our study, and we would like to investigate this problem in a future study. Second, although more than 800 participants were included in the present analysis, the study population may not be representative of the general population because participants were recruited from only one area of Japan. Third, this is a cross-sectional study, so we could not clarify the natural history of paraspinal muscle degeneration and any causal associations between LBP and anthropometric measurements. The Wakayama Spine Study is a longitudinal survey, thus further progress will help elucidate the natural history and any causal associations. Fourth, the influence of level of physical activity was not considered. Individuals with higher activity would be expected to have less fatty infiltration. Fifth, the definition of LBP is different among many studies [29], and the observed association between paraspinal muscle degeneration and LBP might change depending on the definition. We defined LBP as ‘‘LBP present on most days during the past month, in addition to now” based on previous reports [25–29]. Sixth, the influence of physique was not taken into account. Generally, height and body weight correlate with muscle mass. Taller individuals have longer bones and muscles and heavier individuals require more muscle mass for movement, so they would be expected to have greater muscle mass [13].

## Conclusions

We measured the CSA and FI % of the paraspinal muscles (erector spinae, multifidus, and psoas major) using MRI in a Japanese population of individuals ranging in age from 19 to 93 years-old. Our study showed an age-related decrease in CSA and increase in FI % in all muscles in both genders, and that the patterns of the age-dependent degeneration were different according to gender and type of muscles. These measurements of CSA and FI % of the paraspinal muscles may be used as reference values for future comparative studies. Furthermore, our study showed a significant association between FI % of the erector spinae in the upper lumbar spine and LBP. Further investigations along with continued follow-up surveys will continue to confirm the natural history of the paraspinal muscles and their association with clinical symptoms of the lumbar spine.
